# Host specialization of the blast fungus *Magnaporthe oryzae* is associated with dynamic gain and loss of genes linked to transposable elements

**DOI:** 10.1186/s12864-016-2690-6

**Published:** 2016-05-18

**Authors:** Kentaro Yoshida, Diane G. O. Saunders, Chikako Mitsuoka, Satoshi Natsume, Shunichi Kosugi, Hiromasa Saitoh, Yoshihiro Inoue, Izumi Chuma, Yukio Tosa, Liliana M. Cano, Sophien Kamoun, Ryohei Terauchi

**Affiliations:** Iwate Biotechnology Research Center, Kitakami, Iwate Japan; The Sainsbury Laboratory, Norwich Research Park, Norwich, UK; Graduate School of Agricultural Science, Kobe University, Kobe, Japan; The Genome Analysis Centre, Norwich Research Park, Noriwich, UK; John Innes Centre, Norwich Research Park, Norwich, UK; Department of Plant Pathology, Indian River Research and Education Center, University of Florida, Fort Pierce, USA

**Keywords:** *Magnaporthe oryzae*, Pathogenomics, Host specialization, Functional redundancy, Transposable elements, Evolution

## Abstract

**Background:**

*Magnaporthe oryzae* (anamorph *Pyricularia oryzae*) is the causal agent of blast disease of Poaceae crops and their wild relatives. To understand the genetic mechanisms that drive host specialization of *M. oryzae*, we carried out whole genome resequencing of four *M. oryzae* isolates from rice (*Oryza sativa)*, one from foxtail millet (*Setaria italica*), three from wild foxtail millet *S. viridis,* and one isolate each from finger millet (*Eleusine coracana)*, wheat (*Triticum aestivum)* and oat (*Avena sativa)*, in addition to an isolate of a sister species *M. grisea*, that infects the wild grass *Digitaria sanguinalis*.

**Results:**

Whole genome sequence comparison confirmed that *M. oryzae Oryza* and *Setaria* isolates form a monophyletic and close to another monophyletic group consisting of isolates from *Triticum* and *Avena*. This supports previous phylogenetic analysis based on a small number of genes and molecular markers. When comparing the host specific subgroups, 1.2–3.5 % of genes showed presence/absence polymorphisms and 0–6.5 % showed an excess of non-synonymous substitutions. Most of these genes encoded proteins whose functional domains are present in multiple copies in each genome. Therefore, the deleterious effects of these mutations could potentially be compensated by functional redundancy. Unlike the accumulation of nonsynonymous nucleotide substitutions, gene loss appeared to be independent of divergence time. Interestingly, the loss and gain of genes in pathogens from the *Oryza* and *Setaria* infecting lineages occurred more frequently when compared to those infecting *Triticum* and *Avena* even though the genetic distance between *Oryza* and *Setaria* lineages was smaller than that between *Triticum* and *Avena* lineages. In addition, genes showing gain/loss and nucleotide polymorphisms are linked to transposable elements highlighting the relationship between genome position and gene evolution in this pathogen species.

**Conclusion:**

Our comparative genomics analyses of host-specific *M. oryzae* isolates revealed gain and loss of genes as a major evolutionary mechanism driving specialization to *Oryza* and *Setaria*. Transposable elements appear to facilitate gene evolution possibly by enhancing chromosomal rearrangements and other forms of genetic variation.

**Electronic supplementary material:**

The online version of this article (doi:10.1186/s12864-016-2690-6) contains supplementary material, which is available to authorized users.

## Background

*Magnaporthe oryzae* causes disease on an array of important crops such as rice, wheat, barley, finger millet, foxtail millet, and wild grasses [1]. *M. oryzae* has received considerable attention as the causal agent of rice blast disease [2], which leads to 10–30 % loss of harvest per year [3]. *M oryzae* is categorized into several host-specific subgroups that are pathogenic on a variety of plants that include *Oryza* spp., *Setaria* spp., *Triticum* spp., *Avena* spp., *Eleusine* spp. etc. [1, 4, 5]. As with some fungal pathogens *M. oryzae* reproduction is predominantly asexual. When environmental conditions are conducive, *M. oryzae* generates an abundance of asexual clones that rapidly colonize its host. The rapid spread of such asexual pathogens poses a significant threat to global food security, human health and biodiversity [6, 7].

Sexual reproduction for *M. oryzae* is limited to specific geographic regions such as Yunnan, China and India [8–10]. The shift from sexual to asexual reproduction is considered to drive an evolutionary impasse [11]. For instance, linkage between nucleotide positions at each chromosome prevents purifying selection from removing newly arising deleterious mutations. This elevates the accumulation of these deleterious mutations, thereby reducing fitness in a given environment. However, a recent comparative genomic study of asexual pathogens revealed chromosomal rearrangements as a major mechanism for host-specific adaptation [12]. In fungi and oomycete pathogens, these chromosomal rearrangements occur frequently in transposon-rich regions, lineage specific regions and sub-telomeric regions that are enriched for effector genes [13–16]. Effector proteins secreted by pathogens alter the physiology of host plants and enhance colonization by pathogens, often determining the success or failure of infection. Through chromosomal rearrangements effector genes can be gained or lost, diversifying the effector repertoire even in the absence of sexual reproduction [12, 17].

For *M. oryzae*, whole-genome comparative analysis between two *Oryza* isolates P131 (from Japan) and Y34 (from China) revealed frequent chromosomal rearrangements, resulting in structural variation such as gain and loss of genes [18]. The genomic location of the avirulence effector *AVR-Pita* was more variable among asexual rice infecting isolates compared with other cereal infecting isolates, suggesting that selection pressures acting from its cognate resistance gene *Pita* of rice may trigger the diversity in the genome location of *AVR-Pita* [15]. This observation evokes the possibility that the frequency of chromosomal rearrangements may somehow be associated with selection pressure exerted by resistant hosts. In addition, nucleotide substitution is also a potent mechanism for effector diversification [19, 20]. For instance, alleles of *AVR-Pik* that differ in just a few amino acids interact with different specificities with alleles of the resistance gene *Pik* [21, 22].

To date, the evolutionary mechanisms that drive adaptation of *M. oryzae* to specific hosts remain unclear. It has been proposed that *Oryza*-specific isolates of *M. oryzae* are most closely related to those identified on *Setaria* [4]. Whereas the host shift/jump to rice for *M. oryzae* is considered to have happened less than 10,000 years ago following rice domestication [1], *M. oryzae* on *Triticum* was only discovered in Brazil in the late 20th century [23]. This time difference in host shift/jump may have generated distinct patterns of genetic variation between these *M. oryzae* sub-populations. Almost all *Oryza* isolates apart from the center of origin propagate asexually and are only found on cultivated rice (*O. sativa)* [24]. This makes these isolates particularly vulnerable to artificial selection that resulted from breeding resistant rice cultivars as *Oryza* isolates cannot infect any other host plant. In contrast, *M. oryzae* isolates that are adapted to wild *Setaria* spp. have the same host range as isolates from cultivated foxtail millet *S. italica* [25], suggesting that *Setaria* isolates can escape the artificial selection of breeding by shifting to wild host species. This difference between *Oryza* and *Setaria* pathogens could influence their genetic variation within these host-adapted *M. oryzae* populations.

To investigate the evolutionary mechanisms that drive *M. oryzae* host adaptation, we reconstructed the genome sequences of representative isolates from *M. oryzae* specifically infecting *Oryza, Setaria, Eleusine, Avena and Triticum* plant species and its sister species *M. grisea*. Host species specificity in *M. oryzae* has been studied by genetic analyses of these representatives of *M. oryzae* [26–28]. In additions, we resequenced another three isolates each from *Oryza* and *Setaria* pathogens to examine genetic polymorphisms within population of these pathogens. Recently, Chiapello et al. 2015 [29] studied molecular evolution of *M. oryz*ae host-specific subgroups. They revealed that most genes were conserved among *M. oryzae* host-specific subgroups, and only a small number of gene families was associated with host specificity [29]. Compared with their studies, we focused on differences in molecular evolutionary pattern between the host-specific subgroups (*Oryza-Setaria* vs. *Avena-Triticum* pathogens) using different approaches. We discovered that genes showing gain/loss and excess of nonsynonymous substitutions usually encoded proteins whose functional domains are present in multiple copies in each genome. We identified a higher degree of presence/absence polymorphisms for *Oryza* and *Setaria* infecting *M. oryzae* isolates when compared to isolates from *Triticum* and *Avena* even though the genetic distance between *Oryza* and *Setaria* pathogens was smaller than that between isolates from *Triticum* and *Avena*. We also found that the lysin motif (LysM) domain effector gene *Secreted LysM protein 1* (*Slp1*) was lost in the tested *Setaria* isolates. These observations suggest that gain and loss of genes in *M. oryzae* may act as a major evolutionary mechanism driving specialization to *Oryza* and *Setaria* host species. We could also link genes showing presence/absence and nucleotide polymorphisms to transposable elements (TEs), suggesting that TEs may trigger chromosomal rearrangements and mutation.

## Results

### Genome assembly of *M. oryzae* host-specific subgroup species and *M. grisea*

We selected four *M. oryzae Oryza* isolates, four *Setaria* isolates, five host-specific subgroup species and its sister species *M. grisea*, a pathogen of *Digitaria* species for full genome sequencing (Additional file 1: Table S1). We performed de novo assembly for one representative from each host-specific subgroup of *M. oryzae* (Ina168, GFSI1-7-2, Br48, Br58 and Z2-1) and *M. grisea* Dig41 (Table 1). The total assembly size (38.0–40.7 Mbp), N50 (64.1–97.5 Kbp) and number of predicted gene models (12,363–12,671) of *M. oryzae*, were similar to those of previously sequenced genomes of *M. oryzae* 70-15 [2], P131 and Y34 [18], but were smaller than *M. oryzae* host-specifc subgroups in Chiapello et al. 2015 (13,571–14,781) [29]. To further investigate the assembled gene space we performed CEGMA analysis (Additional file 1: Table S2) to assess the completeness of 458 core eukaryotic protein genes (CEGs) in each genome assembly [30]. We used the published genome sequences of *M. oryzae* 70-15 strain [2] and P131 isolate [18] for comparison. The 248 CEGs are classified into four groups [30]. Group1 indicates the least conserved genes of the 248 CEGs. Group number increases with the increasing degree of conservation. The percentage of CEGs for *M. oryzae* host-specific isolates excluding Br48 was consistent with that of *M. oryzae* 70-15 and P131. However, the *M. oryzae* Br48 and *M. grisea* Dig41 isolates showed a lower percentage of CEGs in Group1 and Group3 compared with the controls although these percentages were still comparable to those reported for other published fungal genome sequences [31, 32].

**Table 1:** Summary of de novo assembly and gene prediction

				Isolates				
Isolate	Ina168	Y34^a^	P131^a^	GFSI1-7-2	Br48	Br58	Z2-1	Dig41
Number of corrected short reads (Million reads)	57.6			60.7	56.6	53.5	61.0	60.1
Total size (Gbp)	4.32			4.55	4.24	4.01	4.58	4.51
k-mer size	37			38	39	35	41	40
Number of contigs (> = 0.5 Kbp)	1811			1910	2082	2057	2048	3970
N80 (Kbp)	22.7			30.0	30.3	31.3	25.3	10.1
N50 (Kbp)	77.1			88.3	97.5	91.1	64.1	24.3
Max contig length (Kbp)	400.0			393.6	475.6	640.2	370.9	148.9
Total assembly size (Mbp)	38.0			39.1	40.7	40.2	39.5	41.3
Total number of genes	12,486	12,461	12,363	12,468	12,671	12,626	12,383	11,457
Number of completed genes	11,910	12,106	11,897	11,866	12,020	12,009	11,925	9204
Number of genes with the start codon	12,013	12,110	11,959	11,954	12,071	12,058	11,897	10,139
Number of predicted secretomes	1747	1724	1723	1770	1768	1746	1793	1309

^a^Y34 and P131 data are from Xue et al. [18]

SignalP was performed for genes with the start codon. We did not perform SignalP for genes without the start codon

**Fig. 1:**
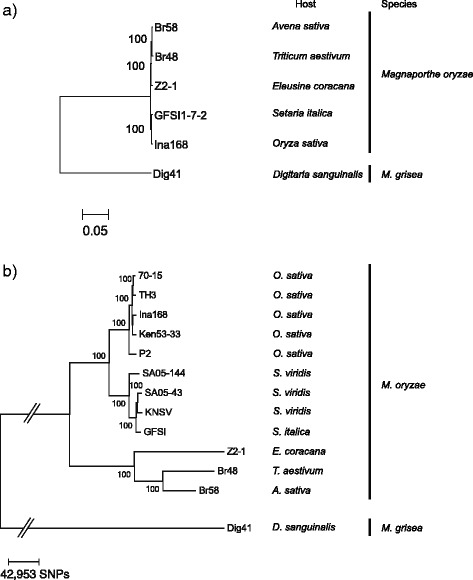
Phylogenetic relationship for *Magnaporthe oryzae* infecting crops and *M. grisea*. **a** The maximum likelihood (ML) tree based on third codon positions of 3257 single copy genes. **b** The ML tree based on 859,067 SNPs distributed on the whole genome. *M. grisea* (Dig41) was used as an outgroup. The numbers on the branches indicate bootstrap probability. The right of the tree show their corresponding host species. The bar below the tree shows genetic distance per site

To classify orthologous and paralogous relationships among the gene models of *M. oryzae* and *M. grisea* isolates, we performed OrthoMCL analysis [33] on all *M. oryzae* isolates and *M. grisea* Dig41 in addition to the published gene models of 70-15 strain, Y34 isolate and P131 isolate. We obtained 13,274 orthogroups, 7845 of which are single copy orthologs in our tested isolates. The number of single copy orthologs is larger than that (6976) in Chiapello et al. 2015 [29]. This discrepancy may be caused by differences in the method of gene prediction. After removing proteins encoded by genes that lacked start and/or stop codons and those with ambiguous orthologous relationships, 3257 single-copy orthologs were subsequently used in the phylogenetic analysis. To investigate the evolutionary relationships among *M. oryzae* and *M. grisea*, we generated a phylogenetic tree based on the third codon position of the single orthologous gene sets using a Maximum likelihood model (Fig. 1a). Since nucleotides in the third codon position are supposed to be under lower selection pressure than those in other codon positions, we expect the tree topology is more reflective of the evolutionary divergence among the isolates. The branch length corresponds to genetic distance based on nucleotide substitutions per site. This tree reconfirmed that *M. grisea* is genetically divergent from *M. oryzae* [1, 5, 34, 35]. Furthermore, the *M. oryzae* isolates from *Oryza* and *Setaria* grouped within a single clade that was clearly separated from the clade containing *M. oryzae* isolates from *Eleusine*, *Triticum* and *Avena. Avena* isolate was closer to *Triticum* isolate than to *Eleusine* isolate. This result is consistent with the previous phylogenetic analyses [1, 5, 29].

To confirm the observed phylogenetic relationship, we aligned the genomic reads from all *M. oryzae* isolates including additional isolates from *Oryza* and *Setaria* to the 70-15 reference genome [2], and constructed a phylogenetic tree based on all the single nucleotide polymorphisms (SNPs) that were estimated from the alignments. The breadth of coverage for the 11 *M. oryzae* isolates ranged from 90.8 to 98.6 % and the read depth ranged from 16 to 78 fold (Additional file 1: Table S3). Using these alignments, we identified a total of 859,067 sites that introduced a SNP in at least one isolate. These 859,067 SNP sites were used to construct a phylogenetic tree of the 11 *M. oryzae* isolates and *M. grisea* Dig41 using a maximum likelihood (ML) model (Fig. 1b). The tree topology was consistent with that of the tree based on the third codon positions of single-copy orthologous genes (Fig. 1a), with the *Oryza* isolates clearly separated from the *Setaria* isolates.

### Gene loss and gain predominantly occurred during host specialization in the *Oryza*-specific subgroup of *M. oryzae*

**Fig. 2:**
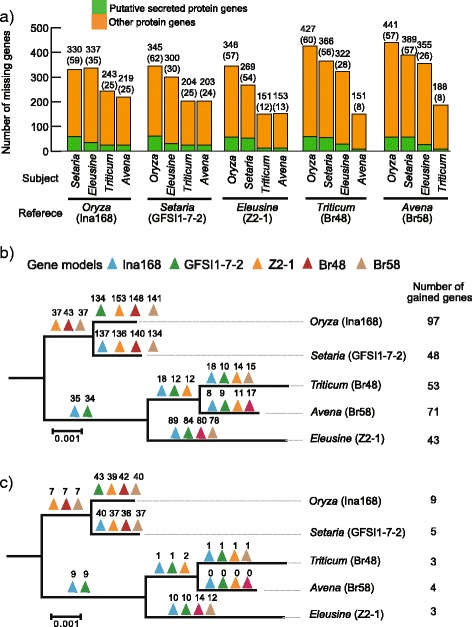
The gain and loss of genes in the five representatives of *Magnaporthe oryzae* host-specific subgroups. **a** The bar plot shows the number of missing genes from each reference genome. The number of secreted protein genes is shown in parentheses. **b** The event of gain and loss of genes on the evolutionary history of the five representatives of *M. oryzae*. **c** The event of gain loss of putative secreted protein genes. The triangles indicate deletion events for each gene model of the host-specific subgroups of *M. oryzae*. The numbers above the triangles are corresponding to the number of genes that was lost once in the evolutionary history of *M. oryzae*. Blue, green yellow, red and ivory indicate the gene models of *Oryza* isolate Ina168, *Setaria* isolate GFSI1-7-2, *Eleusine* isolate Z2-1, *Triticum* isolate Br48, and *Avena* isolate Br58, respectively. At the right of the tree, the number of gain of genes for each host-specific subgroup was shown

For *M. oryzae* isolates from *Oryza*, gain and loss of avirulence effector genes is one of the major mechanisms to avoid effector-triggered immunity of host rice cultivars [19, 20]. However, the contribution of gain and loss of genes in driving host specificity remains unclear. To evaluate this for five representative isolates of *M. oryzae* infecting *Oryza*, *Setaria*, *Eleusine, Triticum*, and *Avena*, we aligned the reads from each isolate to the genome assemblies of all other isolates to determine the breadth of coverage for each gene (Additional file 2). Genes with zero breadth of coverage were considered as absent in a particular pair-wise comparison [36]. Of all the genes, 1.2–3.5 % (151–441) were identified as absent in different comparisons (Fig. 2a). Overall the loss of genes appears to have more frequently occurred in the *M. oryzae* lineages that infect *Oryza* and *Setaria*, with the same tendency reflected in analysis of the predicted secretome. Specially, the *Oryza* Ina168 isolate lost the largest number of genes (345–441) from the reference genomes of *Setaria*, *Eleusine*, *Triticum* and *Avena* isolates.

To further investigate the role of presence/absence polymorphisms in host specialization, we considered genes that were uniquely present or absent in each lineage of the host-specific subgroups of *M. oryzae*. We assigned loss and gain of genes into three categories: (1) genes that were lost once in the evolutionary history of *M. oryzae*, (2) genes that were subjected to multiple deletion events from different processes such as horizontal gene transfer and recombination, and (3) genes that uniquely existed in one host-specific subgroup. The number on each branch of the tree indicates the number of genes belonging to the first category (Fig. 2bc). The genes in the third category were treated as gained genes in each lineage of the host-specific subgroups. Remarkably, more deletion events occurred after the separation of isolates pathogenic on *Oryza* and *Setaria* (134–153) as compared to those of *Triticum* and *Avena* (8–18) although the genetic distance per synonymous site between the *Oryza* and *Setaria* isolates (0.0027 ± 0.0001) was smaller than that between the *Triticum* and *Avena* isolates (0.0053 ± 0.0000). This tendency was more prominent when considering genes encoding putative secreted proteins (Fig. 2c and Additional file 3: Figure S2); 36 to 43 genes were lost for isolates from *Oryza* and *Setaria* compared to 0 to 1 uniquely lost for isolates pathogenic on *Triticum* and *Avena*. In addition, *M. oryzae* isolates from *Oryza* gained the largest number of genes (Fig. 2bc, Additional file 3: Figure S1 and Figure S2), although the number of gained genes did not have such large difference than that of absent genes among the host-specific subgroups.

### Loss of genes is independent of divergence between *M. oryzae* isolates unlike nonsynonymous nucleotide substitutions

**Fig. 3:**
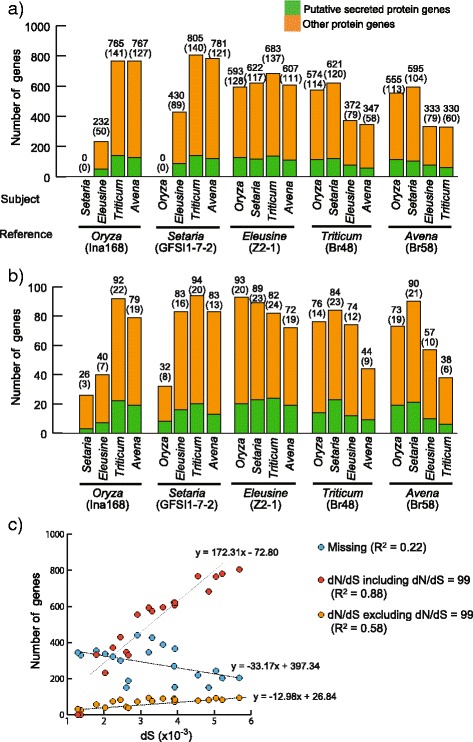
A contrast pattern between nucleotide substitutions and gain/loss of genes in the host-specific subgroups of *Magnaporthe oryzae*. **a**-**b** The bar plot shows the number of genes showing outlier values of dN/dS between the reference isolate and each of other four representatives when dN/dS = 99 was included (**a**) or excluded (**b**). The green and orange bars are corresponding to secreted protein genes and non-secreted protein genes, respectively. The number of secreted protein genes is shown in parentheses. **c** The plot between the number of polymorphic genes and the number of synonymous substitutions per site. The red and blue circles indicate missing genes and genes showing dN/dS > 1.5, respectively. Each of the trend lines is shown

Nucleotide substitutions can contribute to host specialization [37]. To determine the extent of nucleotide changes during host specialization of *M. oryzae* isolates, we estimated the ratio of nonsynonymous substitutions to synonymous substitutions (dN/dS), which is commonly used as a parameter of positive selection (Additional file 2). Genes encoding proteins contributing to host adaptation tend to show a dN/dS ratio over one [36, 37]. At first, we estimated thresholds of dN/dS for detecting outliers in each comparison between the representative isolates when genes with dN/dS = 99 (dS = 0 and dN > 0) were included (Additional file 1: Table S4) or excluded (Additional file 1: Table S5). Genes with outlier ratios of dN/dS are potential fast-evolving genes. As observed in *M. oryzae* effector *AVR-Pik* [21, 22], genes with dN/dS = 99 may be involved in interaction with host molecules and subjected to positive selection. On the other hand, a gene with one nonsynonymous substitutions is likely to have experienced different selection pressures compared to a gene with a larger number of nonsynonymous substitutions, although both genes would show dN/dS = 99 [38]. Given this issue, we reported results including/excluding genes with dN/dS = 99. Since thresholds of dN/dS were less than one in most of pairwise comparisons between the representative isolates when genes with dN/dS = 99 were excluded (Additional file 1: Table S5), dN/dS = 1.5 was used as a threshold value. When genes with dN/dS = 99 were included, 0–6.5 % (0–805) of all the genes showed outlier values of dN/dS in different comparisons (Fig. 3a). When genes with dN/dS = 99 were excluded, genes with outlier values of dN/dS added to 0.2–0.8 % (26–94) of all the genes (Fig. 3b), a proportion that is much smaller than when genes with dN/dS = 99 were included. However, both of the cases showed similar tendency in the proportion of genes with outlier values of dN/dS. When we compared the *Oryza* isolate Ina168 and *Setaria* isolate GFSI1-7-2 to *Triticum* (Br48) and *Avena* (Br58) isolates, a higher proportion of genes showed outliers of dN/dS. When the *Triticum* isolate Br48 and *Avena* isolate Br58 were compared to *Oryza* (Ina168) and *Setaria* (GFSI1-7-2) isolates, a higher proportion of genes showed outliers of dN/dS.

To examine how this compared to the presence/absence analysis, we plotted the number of genes with outlier values of dN/dS when genes with dN/dS = 99 were included or excluded or number of genes identified as absent in the reference genome against the genetic distance at synonymous sites (dS), which is proportional to time from separation for the two pathogen isolates (Fig. 3c). The number of genes with outlier values of dN/dS value was positively correlated with dS, regardless of inclusion or exclusion of genes with dN/dS = 99. However, the number of absent genes was not correlated with the dS values. This indicates that loss of genes was independent of divergence between *M. oryzae* isolates, but the accumulation of nonsynonymous substitutions was dependent on divergence time.

To identify genes that may have specifically evolved in each host-specific subgroup, we categorized genes that showed an outlier dN/dS into 15 patterns (Additional file 3: Figure S3A). The patterns 1 to 4 represent that genes may have rapidly evolved in a specific pair of the representative isolates and were potentially involved in specialization in one isolate of the pair or both isolates. The Venn diagrams in Additional file 3: Figure S3 and Figure S4 were described based on these 16 patterns. A larger number of genes with outlier values of dN/dS was shared between phylogenetically close subgroups. For instance, *Oryza* isolate Ina168 shared 119 genes with *Setaria* isolate GFSI1-7-2, but only 39 genes with *Triticum* isolate Br48 in the Z2-1 gene model (Additional file 3: Figure S3D). Genes encoding putative secreted proteins showed similar patterns (Additional file 3: Figure S3).

### Redundancy of functional domains in the genome promotes genetic variation in host-specific subgroups of *M. oryzae*

**Fig. 4:**
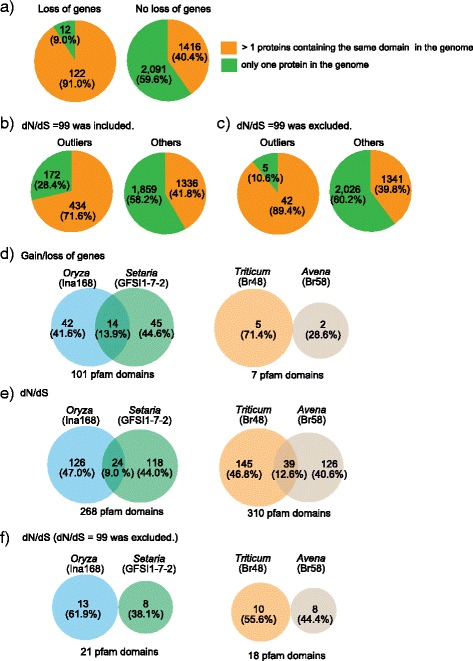
Characterization of genes showing gain/loss and excess of nonsynonymous substitutions. **a**-**c** The pie charts indicate the number of proteins whose domains were detected more than once or only once in the genome in four categories. The high percentage of genes experiencing loss (**a**) and with outlier values of dN/dS when dN/dS = 99 was included (**b**) or excluded (**c**) encodes proteins whose domains are present in multiple copies in each genome. **d** The percentage of shared domains of proteins whose genes showing presence/absence polymorphisms between two host-specific subgroups. **e**-**f** The percentage of shared domains of proteins whose genes showing outlier values of dN/dS between two host-specific subgroups when dN/dS = 99 was included (**e**) or excluded (**f**). The numbers in **(d-f)** are corresponding to the number of pfam domain. The values in (**a**-**f**) are shown when Z-1 gene model was used

Gene deletion or loss of protein function via mutation is deleterious for pathogens if the proteins are essential for development, infection, virulence and/or protection from abiotic and biotic stress. Therefore, the impact of loss of genes and nonsynonymous substitutions to fitness should be different between functionally redundant and non-redundant genes. To test whether this principle was true for the observed variation in the host-specific subgroups of *M. oryzae,* first, we classified functional domains for the predicted proteins using Hidden Markov Model (HMM) analysis against the Pfam database (Additional file 2) [39]. Then, we categorized functional domains into two groups where: (1) domains were detected only once in the genome or, (2) domains were detected more than once in the genome. As expected, *M. oryzae* preferentially lost genes (Fig. 4a) or gained nonsynonymous mutations in genes whose domains are present in multiple copies in the genome (Fig. 4b and c).

To further investigate genes that may be involved in host adaptation, we examined the functional domains identified in proteins that were encoded by genes that were subject to isolate specific loss and/or had an outlier value of dN/dS in one specific pair of the representative isolates. Only a small number of domains (0–13.9 %) were shared between proteins independently lost for each lineage in the host-specific subgroups (Fig. 4d). Similar results were obtained when considering proteins encoded by genes with outlier values of dN/dS regardless of inclusion (Fig. 4e) or exclusion of genes with dN/dS = 99 (Fig. 4f). These results indicate that deletions and nonsynonymous substitutions occurred in genes encoding functionally different proteins after the separation from each of their common ancestors. We could not find statistical enrichment of specific functional domains in the proteins uniquely lost and evolving for each pathogen subgroup.

### Highly polymorphic effectors identified between and within the host-specific subgroups of *M. oryzae*

**Fig. 5:**
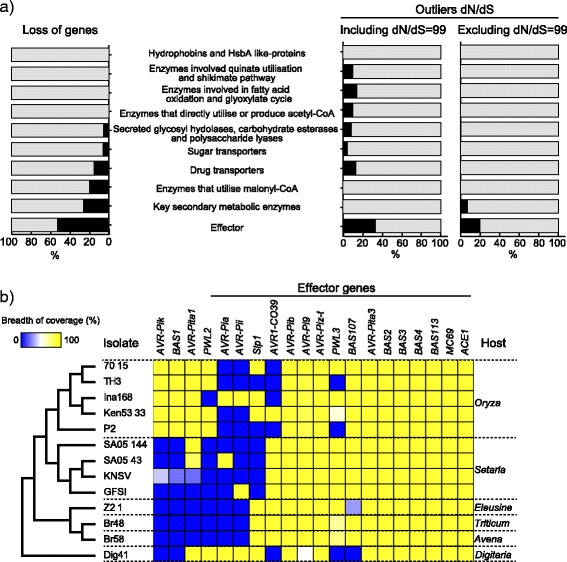
Highly polymorphic in the effector genes between and within the host-specific subgroups of *M. oryzae*. **a** The percentage of genes showing loss, outlier values of dN/dS when dN/dS was included or excluded for each functional category. The black and gray bars indicate the percentage of genes showing loss or outlier values of dN/dS and the others, respectively. These percentages were calculated when we used 70-15 strain genome as the reference. **b** Distribution of presence and absence of genes encoding the known effectors in *M. oryzae* and *M. grisea*. Heat map shows breadth coverage of genes. The blue and yellow panels indicate absence and presence polymorphisms, respectively. The tree indicates the relationship among the tested pathogens based on Fig. 1b

To test how many genes encoding proteins shown to be involved in various stages of appressorium development and infection showed loss among host-specific subgroups of *M. oryzae,* which are absent from at least one of the subgroups, we sorted genes based on the 70-15 strain published gene models [2] and gene categorization [40] (Fig. 5a). As expected from previous studies [18, 19], effector genes showed most frequent loss (52.9 %). In contrast, secreted glycosyl hydrolase, carbohydrate esterases and polysaccharide lyases were not frequently lost (5.7 %), which is consistent with the observation in Chiapello et al. 2015 [29]. Interestingly, ~20 % of genes encoding enzymes that utilize malonyl-CoA and key secondary metabolic enzymes showed presence/absence polymorphisms, contrasting with other enzymes that were monomorphic. This result is different from the conclusion based on clustering of orthologous genes with OrthoMCL [33], in which secondary metabolism genes were highly conserved. OrthoMCL used protein sequences, while our study estimated presence/absence polymorphisms based on the breadth of coverage of DNA sequence reads over genes. Being more sensitive to nucleotide differences, our method tends to detect more presence/absence polymorphisms. We also investigated genes showing dN/dS between the 70-15 strain and each isolate of the host-specific subgroups. Except for genes encoding Hydrophobins and Hydrophobic surface binding protein A (HsbA) like-proteins, Enzymes that utilise malonyl-CoA, and Key secondary metabolic enzymes, all the tested categories included genes with an outlier value of dN/dS when genes with dN/dS = 99 were included (Fig. 5a). Furthermore, 33.3 % of genes encoding effectors showed outlier values of dN/dS. When genes with dN/dS = 99 were excluded, genes with outlier values of dN/dS were detected only in effectors and key secondary metabolic enzymes.

We also specifically tested presence/absence of previously identified effector genes [41] *AVR-Pia*, *AVR-Pii*, *AVR-Pik* [19], *PWL2* [42], *PWL3* [43] *AVR-Pita1* [44], *AVR-Pita3* [45], *AVR-Piz-t* [46], *AVR1-CO39* [47], *BAS1*-*4* [48], *BAS107*, *BAS113* [49], *AVR-Pi9* [50], *AVR-Pib* [51], *S1p1* [52], *MC69* [53], *ACE1* [54] in *M. oryzae* representative isolates together with the *M. grisea* Dig41 isolate (Fig. 5b). In addition, presence/absence of the effector genes in three *Oryza* isolates and three *Setaria* isolates, which were re-sequenced for the analysis of genetic polymorphisms within these isolates, was also tested. The effector genes in *M. oryzae* were highly polymorphic in isolates infecting *Oryza* and *Setaria. AVR-Pita1, AVR-Pia* and *AVR-Pii* were missing in isolates infecting other cereals, but were present in *M. grisea* isolate Dig41. Interestingly, *S1p1*, an effector containing a LysM domain [52] was lost in all tested *Setaria* isolates and was polymorphic in the *Oryza* isolates, suggesting that *Slp1* might be associated with host specialization.

### Lower nucleotide diversity but more frequent nonsynonymous mutations for *M. oryzae* isolates pathogenic on *Oryza* spp

Although *Oryza* and *Setaria*-specific subgroups are not separate species, the estimation of intra-specific variation is useful to test how different host environments have shaped genetic variations in *M. oryzae*. The representative isolate and the three additional isolates of each subgroup were used for the analyses of intra-specific variation. At first, to address gain and loss of genes involved in host specialization in the *Oryza* and *Setaria*-specific subgroups, we identified presence and absence polymorphisms in each population of *M. oryzae* on *Oryza* and *Setaria* (Additional file 3: Figure S5). The number of presence/absence polymorphisms in the *M. oryzae Oryza* subgroup was larger than that for the *Setaria* subgroup. This tendency was also more pronounced for genes encoding predicted secreted proteins. These observations were detected in all the gene models estimated from the genome sequences of 70-15 and the representative isolates.

We also calculated nucleotide diversity (π) in *M. oryzae Oryza* and *Setaria-*specific subgroups (Table 2). The isolates pathogenic on *Oryza* spp. contained about half the nucleotide diversity of the isolates pathogenic on *Setaria* spp. for both coding and non-coding regions.

**Table 2:** Summary of nucleotide diversity (π) of *Oryza* and *Setaria* isolates

Group	Gene coding region		Non-coding region
	Synonymous	Nonsynonymous	
*Oryza* pathogen (*n* = 5)	0.00044	0.00019	0.00035
*Sataria* pathogen (*n* = 4)	0.00101	0.00031	0.00073

To further assess the process of DNA polymorphism accumulation between these populations, we applied the McDonald and Kreitman’s (MK) test [55] to the observed polymorphisms in the gene coding regions (Table 3). In the neutral hypothesis of molecular evolution, the ratio of synonymous to nonsynonymous divergences between the populations would be expected to be similar to the ratio of synonymous to nonsynonymous polymorphisms within each population. When the MK test was applied to each gene, none of the tests gave significant results following multiple corrections. On the other hand, when DNA variation in all the tested genes were combined, for both *M. oryzae Oryza* and *Setaria* subgroups, the synonymous/nonsynonymous ratio of DNA polymorphisms levels did not conform to that of divergence (Table 3). In particular, it is notable that there are more SNPs causing nonsynonymous changes (4408) than those causing synonymous changes (3312) within the isolate infecting *Oryza* spp.

**Table 3:** Results of McDonald and Kreitman’s (MK) test applied to *Oryza* and *Setaria* isolates of *M. oryzae*

	Synonymous	Nonsynonymous	Χ^2^	*P*
Fixed differences between *Oryza* isolates and *Setaria* isolates	7631	4940		
Polymorphisms in *Oryza* isolates	3312	4408	609.32	*P* < 2.2e-16
Polymorphisms in *Setaria* isolates	5933	5411	171.15	*P* < 2.2e-16

### Tight linkage between transposable elements and polymorphic genes in *M. oryzae* isolates infecting *Oryza* spp. and *Setaria* spp

**Fig. 6:**
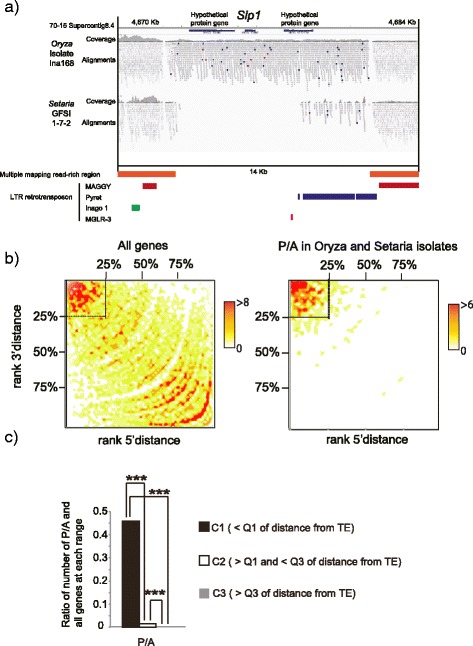
Genes showing presence and absence polymorphisms are linked to transposable elements. **a** The alignments view of short reads around *SLP1* region. *SLP1* was surrounded by multiple mapping reads and partial LTR retrotransposons. **b** Distribution of presence and absence polymorphisms within *Oryza* and *Setaria* isolates according to local TE density. *Magnaporthe* genes were ranked on the basis of ascending distance from 5’-end (y-axis) and 3’-end (x-axis) of genes to TEs. The number of genes corresponding to genes in each bin is shown as a heat map. 25, 50 and 75 % indicate first quartile, median and third quartile, respectively. The left and right panels are the distributions of all genes and genes showing presence/absence polymorphisms, respectively. **c** The bar plot shows the ratio of the number of genes showing presence and absence polymorphisms within *Oryza* and *Setaria* isolates to genes at each of category C1, C2, and C3. Statistical significance was evaluated by using exact Wilcoxon Mann–Whitney rank sum test (***: *P* < 0.001). Since the ratio in the C3 was very small, the value is not seen in the figure

In *M. oryzae*, effector genes displaying presence/absence polymorphisms are often located next to transposable elements (TEs) [15, 18, 19]. For example, *Slp1* of *Oryza* isolate Ina168 resides between two multiple mapping read-rich regions including partial sequences of long terminal repeat (LTR) retrotransposons (Fig. 6a). It is interesting to note that *Slp1* is missing from *Setaria* isolate GFSI1-7-2 but the flanking multiple mapping read-rich regions are conserved (Fig. 6a), suggesting possible excision of *Slp1* in Setaria isolates. Furthermore, TEs are known to contribute to insertion and deletion of DNA segments within the genome and also contribute to duplication of certain regions. To further investigate the relationships between transposable elements and the presence/absence of linked genes, we classified *M. oryzae* genes according to their distance to the closest TEs. Since the distribution of distances between the gene and TE were highly skewed for both 5’- and 3’-directions (Additional file 3: Figure S6), we decided to classify them non-parametrically to the first quartile (C1: genes close to TE, <25 % of the total genes), the last quartile (C3: genes most distant from TE, <25 % of the total genes) and in between (C2: 25–75 %). We observed that genes tightly linked to TEs showed a higher incidence of presence/absence polymorphisms within *Oryza* and *Setaria* isolates (Fig. 6bc).

**Fig. 7:**
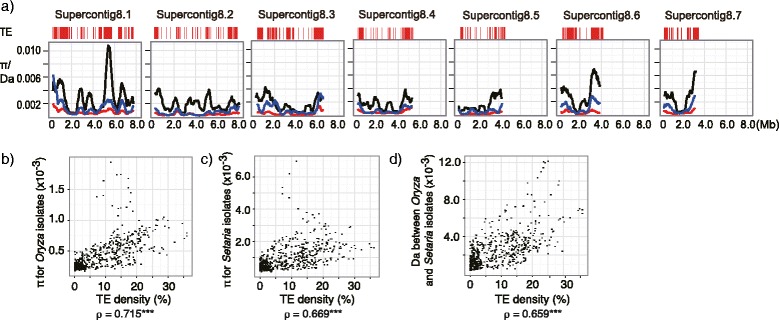
The level of DNA polymorphisms in *M. oryzae* is correlated with the density of transposable elements. **a** Results of sliding window analysis of π for *Oryza* isolates (*red*), π for *Setaria* isolates (*blue*), and the number of net nucleotide substitutions per site (Da) (Nei 1987) between *Oryza* and *Setaria* isolates are given for seven supercontigs. These values were estimated based on nucleotide variations at synonymous sites and noncoding regions. Window size is 500 Kbp. Step size is 50 Kbp. TE: location of transposable elements. Correlation between the density of transposable elements and three measurements: nucleotide diversity (π) of *Oryza* (**b**) and *Setaria* (**c**) isolates, and distance (Da) between *Oryza* and *Setaria* isolates (**d**)

Next, to test whether TEs influence the level of nucleotide substitutions and polymorphisms, we separately calculated nucleotide diversity (π) for *M. oryzae Oryza* and *Setaria* specific subgroups, respectively, genetic distance (*Da*) between *Oryza* and *Setaria* specific subgroups for Supercontigs 8.1 to 8.7 (Fig. 7a). The values of *Oryza* π, *Setaria* π and *Da* were variable along the selected genomic regions for all contigs. There was an obvious correlation among these three values (Fig. 7a, Additional file 3: Figure S7). We also addressed the position of transposable elements (TEs) on the genome and studied the relationship between TE location and values of *Oryza* π, *Setaria* π and *Da*. As seen in Fig. 7a, there was a tendency for genomic regions with a higher density of TE’s to show higher values of *Oryza* π, *Setaria* π and *Da* (Fig. 7bcd). TE density had a statistically significant positive correlation with *Oryza* π, *Setaria* π and *Da*. Note that values of *Oryza* π, *Setaria* π and *Da* were calculated after removing the DNA sequences corresponding to transposable elements. These results suggest that genomic regions close to transposable elements tend to harbor higher levels of nucleotide diversity within populations and larger divergence between populations.

To further investigate the relationships between the transposable elements and DNA polymorphisms of the linked genes, we classified *M. oryzae* genes according to their distance to the closest TE according to the same criteria of the analysis of presence/absence polymorphisms (Fig. 6c). For each class of C1, C2 and C3, the level of presence/absence polymorphisms, nucleotide diversity for synonymous and nonsynonymous DNA polymorphisms and divergence between *Oryza* and *Setaria* pathogens (Da) was examined (Additional file 3: Figure S8). We noted that genes closely linked to TEs (C1) showed significantly higher levels of presence/absence polymorphisms and synonymous and nonsynonymous nucleotide polymorphisms and divergence between *Oryza* and *Setaria* pathogens than the genes distantly located to TEs (C2 and C3). From these results, we conclude that presence of transposable elements may increase genetic diversity and divergence of genes located nearby.

## Discussion

### Higher rates of gene loss in *M. oryzae* isolates from *Oryza*, *Setaria*, and *Eleusine* than in isolates from *Triticum* and *Avena*

The majority of genes were conserved across all tested host-specific subgroups of *M. oryzae* with only a small fraction (1.2–3.5 % of the genes) dynamically gained and lost, as reported in Chiapello et al. 2015 [29]. Previous studies have suggested that the insertion of transposons and the positioning of genes close to telomeres may enhance mobility of genes via chromosomal rearrangements including translocation and deletion [12, 15, 44, 56]. In addition, our study revealed that functional redundancy may contribute to the dynamics of *M. oryzae* genes. Interestingly, we observed that in comparisons between host-specific subgroups, the isolate infecting rice showed the largest number of genes lost from the genome (345–441 genes), followed by the isolate from *Setaria* spp. (269–389 genes) and the isolate from *Eleusine* spp. (300–355 genes), respectively. The number of genes lost from the genomes of *Triticum* and *Avena* pathogens when compared to all other host-specific subgroups was relatively small (ranging from 151 to 243) (Fig. 2a). These observations could be explained by the difference in evolutionary time following the host shift (divergence time). Accordingly, *Oryza*, *Setaria*, and *Eleusine* specific subgroups of *M. oryzae* are predicted to have a longer evolutionary history of host specificity when compared to *Triticum* and *Avena* specific subgroups. The origin of *Oryza M. oryzae* isolates was dated back to rice cultivation ~10,000 years ago [1], whereas *Triticum* isolates were first reported in the 1980s [23], suggesting a recent host jump and specialization. Following host shift and during the ensuing specialization period, deletions in non-essential and/or deleterious genes may have been retained through purifying selection or random genetic drift. Our analysis showed that *M. oryzae* preferentially lost genes whose domains were present as multiple copies in the genome and encoded different functional domains between isolates pathogenic on *Oryza* spp. and *Setaria* spp. In addition, selection pressures may vary between hosts and could also shape the distinct patterns between the host-specific subgroups. Intensive breeding of rice cultivars resistant to blast disease may have been more extensive compared with *Setaria* spp. and *Eleusine* spp. resulting in more frequent gain and loss of genes in *M. oryzae* isolates pathogenic on *Oryza* spp.

### Accumulation of non-synonymous substitutions in the host-specific subgroups of *M. oryzae*

An excess of nonsynonymous mutations compared to synonymous mutations was found in 0–6.5 % of genes in *M. oryzae* when genes with dN/dS = 99 were included. Genes with outlier values of dN/dS could be classified into two groups. One group was potentially under positive selection. Positive selection was common among the effector genes *AVR-Pizt*, *Slp1, PWL3, AVR-Pita3* and *BAS2* (Fig. 5, Additional file 2), some of which were previously reported within *Oryza* isolates [20]. Since the avirulence effectors are considered to have coevolved with their cognate resistance genes of the host plants [57], an arms race with the host plant immune system could explain the excess of nonsynonymous substitutions (e.g. [21, 22]). Of the putative secreted protein genes, 0–1.1 % showed outlier values of dN/dS as observed in these avirulence proteins when genes with dN/dS = 99 were included. These proteins are presumably secreted outside the pathogen and possibly interact with host molecules, contributing to specialization to the host environment as observed in several pathosystems [17, 37].

Another group of genes with outlier values of dN/dS had a few nonsynonymous substitutions and zero synonymous substitutions. Small synonymous divergence is not enough to give statistical power to detect positive selection [38]. The number of genes with outlier values of dN/dS was positively correlated with genetic divergence between host specific subgroups. This observation is consistent with an overall random accumulation of nonsynonymous substitutions in this group and the degree to which these genes are under positive selection cannot be determined with the current data. As detected in the presence/absence polymorphism analysis, these genes generally encode proteins whose domains were present in multiple copies in the genome. Therefore, the observed nonsynonymous substitutions may not have had deleterious effects on the fitness of the pathogen due to the functional redundancy of the proteins targeted for nonsynonymous mutation. It is interesting to note, however, that some avirulence effectors that have no synonymous substitutions have nonsynonymous substitutions that impact their activity and thus in these cases the mutations are probably beneficial and positively selected (e.g. AVR-Pik/Pik, [21, 22]). The degree to which this affects the other genes in this category cannot be determined.

Recently, de Guillen et al. 2015 [58] reported the existence of a family of effectors (MAX-effectors) that has no nucleotide sequence homology but has conserved 3D protein structure. The protein structure of these effector proteins may enable the accumulation of nonsynonymous substitutions involved in the adaptation to various host species. The expansion and diversification of the MAX-effectors that was noted in *M. oryzae* [58] is consistent with our view that gene gain and loss are major drive of evolution in this species. In combination with the protein structure scaffold of the MAX-effectors, relaxation of purifying selection due to functional redundancy may have accelerated protein evolution in *M. oryzae*.

### The LysM domain containing effector *Slp1* was lost in the *M. oryzae* subgroup that specifically infects *Setaria* plants

The effector *BAS1* and *AVR-Pik* are observed in the *Oryza*-specific subgroup and not in the *Setaria*-specific subgroup. These effectors have been shown to be specifically induced during the biotrophic phase of *M. oryzae* infection on rice [48, 59]. This suggests they may contribute towards specialization of this *M. oryzae* subgroup to rice plants. On the other hand, the LysM domain effector gene *Slp1* was missing in two *Oryza* isolates (TH-3 and P2) and in the *Setaria* subgroup. The Slp1 is an apoplastic effector that binds Chitin and contributes to the avoidance of pathogen-associated molecular pattern (PAMP) triggered immunity [52]. Furthermore, knocking out of Slp1 reduced pathogenicity of *M. oryzae* on rice plants [52]. The 70-15 strain had eight putative secreted proteins containing LysM domains and the *Oryza* isolate Ina168 had one additional putative secreted protein containing a LysM domain. Furthermore, the Setaria-specific subgroup lost Slp1 but harbor an extra LysM domain-containing protein, which potentially could compensate for the lack of Slp1. The loss of Slp1 from the Setaria-specific subgroup could be due to (1) functional redundancy driven by genetic drift, or (2) Slp1 being deleterious for adapting *Setaria* plants driving loss by purifying selection. As all other crop-infecting *M. oryzae* and also *M. grisea* Dig41 maintain *Slp1*, this suggests an essential role for the effector, with its loss in Setaria-specific subgroup likely a result of host specialization*.* However, we only examined a limited number of *Setaria* isolates. To challenge this hypothesis, it is necessary to survey a larger number of *Setaria* isolates to estimate the effector frequency in population of *Setaria* isolates.

### Linkage to transposons is associated with elevated mutation rates

TEs are known to contribute to insertion, deletion and duplication of DNA segments in a genome. We observed that genes tightly linked to TEs show a higher incidence of presence/absence polymorphisms (Fig. 6). In addition to this effect, we also observed a higher level of nucleotide polymorphisms in the genes more closely linked to TEs than those distantly linked to TEs (Additional file 3: Figure S8). This observation suggests that TEs can generate higher nucleotide level diversity as well as structural diversity in genomes. In a species like *M. oryzae* that predominantly propagates asexually, genetic recombination by sexual crosses is limited. Asexual propagation limits genetic diversity that is needed for adaptation to new environments and enhances the accumulation of deleterious mutations (Muller’s ratchet) reducing the mean fitness of the species (population). We hypothesize that TEs may substitute the function of sexual recombination to contribute to maintaining genetic diversity. For instance, genes closer to TEs could potentially be inadvertently mutagenized during imperfect TE-insertion mediated mismatch repair, thereby enhancing genetic diversity during prolonged periods of exclusively asexual reproduction.

### Abrupt changes in environment drove accumulation of nonsynonymous mutations and presence/absence polymorphisms in *M. oryzae Oryza* isolates

We identified an excess of nonsynonymous DNA polymorphisms as compared to synonymous ones and presence/absence polymorphisms in the *Oryza*-specific subgroup. Since there is no wild rice species in Japan, Japanese *Oryza* isolates survive only on cultivated rice. In contrast, *Setaria* isolates can survive on wild Setaria species, which are commonly distributed across Japan. Therefore, *Oryza M. oryzae* isolates, are more vulnerable to artificial selection of rice breeding. These different selection pressures could generate more frequent presence/absence polymorphisms in the *Oryza*-specific subgroup even though the level of nucleotide diversity in *Oryza* isolates was smaller than that in *Setaria* isolates. This excess of nonsynonymous polymorphisms in the *Orzya* subgroup could be explained by two general mechanisms. One is positive selection on protein coding genes in the *Oryza*-specific subgroup. For example, *AVR-Pik* seems to be engaged in an arms race with its cognate resistance gene *Pik* of rice [21, 22]. The observed amino acid differences between AVR-Pik isoforms determine binding and activation of corresponding Pik immune receptor isoforms. The other is that this higher level of nonsynonymous polymorphisms in the isolates infecting *Oryza* spp. may be caused by relaxation of purifying selection by its smaller population effective size. In either mechanism, the relatively higher level of nonsynonymous DNA polymorphisms in the *Oryza*-specific subgroup may provide the genetic and phenotypic diversity that becomes favorable when the population encounters abrupt changes of environment, e.g. changes in host cultivars. Such high level of intraspecific genetic plasticity should facilitate the persistence of this pathogen lineage despite the constantly changing host environment.

## Conclusions

This comparative genomics study sheds light on the evolutionary mechanisms underpinning *M. oryzae* specialization to host plant species. We investigated gain and loss of genes and nucleotide substitutions between host-specific subgroups of *M. oryzae.* We found that redundancy of functional domains in the genome may have enabled a high degree of intra-specific gain and loss of genes and accumulation of nonsynonymous substitutions. Interestingly, these two mechanisms of genetic variations showed contrasting patterns in the comparisons of the host-specific subgroups (*Oryza-Setaria* vs. *Avena-Triticum* pathogens). The number of presence/absence of genes was not correlated with genetic divergence between the host specific subgroups, while the number of genes with outlier values of dN/dS correlated well with genetic distance. Though the genetic distance between the isolates infecting *Oryza* and *Setaria* was smaller than that between those infecting *Triticum* and *Avena*, the loss and gain of genes in *Oryza* and *Setaria* isolates were more frequently observed than those in *Triticum* and *Avena* isolates. Considering that *Oryza* and *Setaria* specific subgroups of *M. oryzae* have a longer evolutionary history of host specificity when compared to *Triticum* specific subgroup, this observation suggests that the differences are not explained by the elapsed time of genetic isolation but rather by coevolution with the specific host. In addition, the study of variations in *Oryza*- and *Setaria*-specific subgroups of *M. oryzae* revealed that the isolates pathogenic on *Oryza* had higher number of nonsynonymous polymorphisms, despite having smaller nucleotide diversity than the isolates pathogenic on *Setaria*. Differences in hosts and frequency in abrupt changes of environments may have shaped the contrasting patterns of genetic variations between these host-specific subgroups of *M. oryzae*.

## Methods

### DNA extraction and sequencing

We extracted DNA from mycelia of *M. oryzae* and *M. grisea*, which are maintained in Laboratory of Plant pathology, Kobe University, Iwate Biotechnology Research Center and National Institute of Agrobiological Sciences Genbank, using the DNeasy Plant Maxi kit (QIAGEN). Five μg of the DNA was used for constructing the paired-end libraries following manufacturer's instructions. The libraries were used for cluster generation on a flow cell and sequenced for 76 cycles on an Illumina Genome Analyzer IIx. Base calling and filtering of low quality bases were performed using sequence control software (SCS) real-time analysis, BCL converter and the GERALD module (Illumina, San Diego, CA, USA).

### De novo assembly of short reads and gene predictions

Adapter sequences were removed from short reads adapter using a software cutadapt [60]. Unpaired reads were filtered using a perl program cmpfastq (http://compbio.brc.iop.kcl.ac.uk/software/index.php). We conducted De novo assembly using a software ABySS [61] according to the pipeline of Saunders et al. 2014 [62]. We evaluated N50 of the assembled sequences that were created following different K-mer values and chose the sequences having the highest N50 value for the further analyses. To assess quality of the assembled sequences, we used the program CEGMA [30]. We predicted genes from the assembled sequences based on *ab-initio* and empirical methods. We estimated gene structure based on the published transcript sequences version 8 of *M. oryzae* 70-15 strain (Broad institute) and known *M. oryza e*ffectors [41], and then created an annotation files as general feature format (gff) using a software exonerate version 2.0 [63]. Genes of each assembled sequence of *M. oryzae* and *M. grisea* isolates were predicted using a software AUGUSTUS version 2.7 [64] after the training based on the created annotation files. SignalP4.0 was performed to predict secreted proteins [65]. Functional domains of the predicted genes were inferred using HMM-based search against Pfam [39]. BLASTN was performed using the predicted proteins as query against Swiss-Prot. Blast2GO [66] was run using the outputs of the BLASTN [67] as input files to clarify descriptions of proteins encoded by the predicted genes. To reveal orthologous relationship for each gene model, OrthoMCL was performed with 95 % identity cutoff [33]. We used third codon positions of 3257 single copy genes having start and stop codons for the construction of phylogenetic trees.

To identify SNPs and indels among *Magnaporthe* species, paired-end short reads from each species were aligned with BWA [68] to the 70-15 strain (MG8) reference sequence and the assembled genome sequences of the five crop infecting *M. oryzae* isolates. Alignment files were converted to SAM files using SAMtools version 0.1.8 [69]. To remove low quality reads, modify misalignment reads and to detect reliable SNPs and short indels, the SAM files are applied to the filter pipeline “Coval” [70]. This pipeline reduces misalignments around short indels, and removes reads with errors arising from low phred-like quality scores and paired-end reads of an insert size longer than expected from the preparation of libraries. We called homozygous SNPs with default setting of Coval. We ignored heterozygous SNPs because we extracted DNAs from haploid mycelia of *Magnaporthe* species. Most of heterozygous SNPs could be caused by misalignments or reads that are aligned on multiple genomic regions.

To detect loss of genes in tested isolates from the reference genome, we estimate breadth coverage over a gene based on the alignments according to Raffaele et al. 2010 [36]. When short reads were aligned to their own assembled genome sequence using BWA, the smallest value of the breadth coverage was from 56 % (Ina168 isolate) to 78 % (Z2-1 isolate). If the breadth coverage is more than 50 %, the tested gene was judged presence on these minimum coverage values. If the breadth coverage is equal to zero percent, the tested gene was judged absence. The other values of breadth coverage were regarded as ambiguity and were not used for evaluating presence and absence polymorphisms. To clarify the pattern of presence and absence polymorphisms among the crop infecting *M. oryzae* isolates, Venn diagram was created using an online diagram software Cleately (Cinergix, Melbourne, Australia).

### Construction of phylogeny and estimation of nucleotide substitutions and polymorphisms

The maximum likelihood trees were constructed using the RAxML software [71]. Consensus sequences for each gene were created based on SNPs and pileup format of SAMtools. We regarded positions where no short reads were aligned as unknown nucleotides. When nucleotides were different from those of the reference genome but were not regarded as homozygous SNPs by Coval, we also treated them as unknown nucleotides. Synonymous divergence (dS) and nonsynonymous divergence (dN), and omega (dN/dS) between *M. oryzae* and *M. grisea* isolates were estimated based on the consensus sequences using yn00 [38], which is an ad hoc counting method. When unknown bases were occupied over 80 % of total length of the consensus sequence, we did not include this consensus sequence for calculation of dN, dS and omega. Thresholds for detecting outliers of dN/dS were calculated by 1.5 × interquartile range + 3^rd^ quartile of dN/dS in each pairwise comparison between isolates. If a threshold value was < 1.5, 1.5 was used as the threshold value.

To examine level and pattern of nucleotide polymorphisms within *Oryza* isolates and *Setaria* isolates, we calculated nucleotide diversity π [72] and the number of net nucleotide substitutions per site between populations Da [72] as a parameter of genetic distance between *Oryza* isolates and *Setaria* isolates by using the Bioperl libraries. Sliding window analysis of π, and Da was performed with the 500 Kbp widow size and 50 Kbp step size. R, a programming language for statistical computing, was used for visualization of the sliding window analysis. To test the neutral hypothesis of molecular evolution, McDonald and Kreitman test [55] was carried out according to the method of DNASP [73].

### Linkage of transposable elements and genetic variations

To test relationship between transposable elements (TEs) and genetic variations, positions of TEs were identified. We performed BLASTN [67] using known *M. oryzae* transposable TEs (Additional file 1: Table S6) as query and the 70-15 genome sequence as database with the default setting. The aligned genome regions were regarded as the positions of TEs. We calculated physical distance from TEs and 5´ or 3´end of tested genes. To test whether genes showing genetic variations within *Oryza* isolates and *Setaria* isolates linked to TEs or not, we used positions of TEs on the supercontigs of *M. oryzae* 70-15 strain.

## Ethics

This article does not contain any studies with human participants or animals performed by any of the authors.

## Consent to publish

Not applicable.

## Availability of data and materials

Nucleotide sequence data reported are available in the DDBJ Sequenced Read Archive under the accession numbers DRR059884-DRR059895. The assembled genome sequences of *M. oryzae* isolates Ina168, GFSI1-7-2, Br48, Br58 and Z2-1 and *M. grisea* isolate Dig41 have been deposited to DDBJ Sequenced Read Archive under the accession numbers DRZ007458, DRZ007459, DRZ007460, DRZ007461, DRZ007462, and DRZ007468, respectively. The annotation files of *M. oryzae* isolates Ina168, GFSI1-7-2, Br48, Br58 and Z2-1 and *M. grisea* isolate Dig41 are available in the DDBJ Sequenced Read Archive under the accession numbers DRZ007463, DRZ007464, DRZ007465, DRZ007466, DRZ007467 and DRZ007469, respectively.

## Additional files

Additional file 1: This file contains Supplemental Tables S1 to S6 and their legends. (PDF 130 kb) Additional file 2: This sheet includes information about annotation, presence/absence polymorphisms and dN/dS for each of the gene models. (XLSX 12390 kb) Additional file 3: This file contains Supplemental Figures S1 to S8 and their legends. (PDF 1373 kb)

## Abbreviations

CEGs: core eukaryotic protein genes
Da: the number of net nucleotide substitutions per site between populations
dN/dS: the ratio of nonsynonymous substitutions to synonymous substitutions
dS: synonymous divergence
dN: nonsynonymous divergence
HsbA: hydrophobic surface binding protein A
LTR: long terminal repeat
LysM: lysin motif
MK test: McDonald and Kreitman’s test
ML: maximum likelihood
PAMP: pathogen-associated molecular pattern
Slp1: Secreted LysM protein 1
SNPs: single nucleotide polymorphisms
TEs: transposable elements
π: nucleotide diversity
