# Supplementary material for: Host specialization of the blast fungus Magnaporthe oryzae is associated with dynamic gain and loss of genes linked to transposable elements
Source: BMC Genomics. 2016 May 18;17:370. doi: 10.1186/s12864-016-2690-6 (PMC4870811; doi:10.1186/s12864-016-2690-6)
Supplement: Additional file 1: — This file contains Supplemental Tables S1 to S6 and their legends. (PDF 130 kb) [file 12864_2016_2690_MOESM1_ESM.pdf]

Table S1. Fungal isolates used for the genome sequencing.

Isolate	Location	Host	Isolation year	Reference
Ina168	Japan (Aichi)	*Oryza sativa*	1958	Kiyosawa 1974
Ken53-33	Japan (Aichi)	*O. sativa*	1953	Kiyosawa 1974
TH3	Thailand	*O. sativa*	unknown	Yoshida et al. 2009 [18]
P-2	Japan (Niigata)	*O. sativa*	1948	Kiyosawa 1974
GFSI1-7-2	Japan (Gifu)	*Setaria italica*	1977	Kato et al. 2000 [1]
KANSV1-4-1	Japan (Kanagawa)	*S. viridis*	1975	Kato et al. 2000 [1]
SA05-43	Japan (Nagasaki)	*S. viridis*	2005	Yamagashira et al. 2008 [23]
SA05-144	Japan (Kyoto)	*S. viridis*	2005	Yamagashira et al. 2008 [23]
Z2-1	Japan (Kagawa)	*Eleusine coracana*	1977	Kato et al. 2000 [1]
Br48	Brazil (Mato Grosso do Sul)	*Triticum aestivum*	1990	Murakami et al. 2000 [45]
Br58	Brazil (Parana)	*Avena sativa*	1990	Takabayashi et al. 2002 [46]
Dig41	Japan (Hyogo)	*Digitaria sanguinalis*	unknown	Kato et al. 2000 [1]

Kiyosawa, S. (1974) Studies on genetics and breeding of blast resistance in rice. Misc. Publ. Natl. Inst. Agric. Sci. 1, 1–58.

Table S2. Quality evaluation of genome assembly using CEGMA for five *Magnaporthe oryzae* isolates and one *M. grisea* isolate

Species	Isolates	% of 248 core eukaryotic genes				Reference
		Group1 66 genes	Group2 56 genes	Group3 61 genes	Group4 65 genes	
M. oryzae	70-15	96.97 (98.48)	98.21 (98.21)	98.36 (100)	100 (100)	Dean et al. 2005 [2]
M. oryzae	P131	96.97 (98.48)	98.21 (98.21)	98.36 (100)	100 (100)	Xue et al. 2012 [17]
M. oryzae	Ina168	96.97 (98.48)	98.21 (98.21)	98.36 (100)	100 (100)	In this study
M. oryzae	GFSI1-7-2	96.97 (98.48)	98.21 (98.21)	98.36 (100)	100 (100)	In this study
M. oryzae	Br58	96.97 (98.48)	98.21 (98.21)	98.36 (100)	100 (100)	In this study
M. oryzae	Br48	95.45 (98.48)	96.43 (98.21)	98.36 (100)	100 (100)	In this study
M. oryzae	Z2-1	96.97 (98.48)	98.21 (98.21)	98.36 (100)	98.46 (100)	In this study
M. grisea	Dig41	84.85 (90.91)	91.07 (92.86)	90.16 (98.36)	100 (100)	In this study

The percentage of the 248 core eukaryotic genes (CEGs) aligned as complete gene is shown. The percentage of the CEGs aligned as partial gene copies is shown in parentheses. Group1 indicates the least conserved genes of the 248 CEGs. Group number increases with the increasing degree of conservation. Group 4 represents the most conserved genes (Parra et al. 2009 Nucleic Acids Res. 37: 289–297.)

Table S3. Summary of whole genome resequencing of 11 *Magnaporthe oryzae* isolates and one *M. grisea* isolate (Dig41)

Isolate	Host	Origin	No. of reads	Total size (Gb)	Coverage (%)	Read depth	No. of SNPs	No. of short indels
Ina168	*Oryza sativa*	Japan	57,632,566	4.32	98.6	78.0	13,395	1,867
Ken53-33	*O. sativa*	Japan	20,722,870	1.55	98.5	23.9	14,103	2,306
P2	*O. sativa*	Japan	41,977,090	3.15	96.1	33.0	25,963	3,852
TH3	*O. sativa*	Thailand	13,657,828	1.02	98.0	16.0	10,137	1,716
GFSI1-7-2	*Setaria italica*	Japan	60,721,550	4.55	96.4	69.6	108,667	9,663
KANSV1-4-1	*S. viridis*	Japan	40,986,824	3.07	94.8	25.4	100,734	9,162
SA05-43	*S. viridis*	Japan	18,998,940	1.42	94.9	17.9	97,563	9,259
SA05-144	*S. viridis*	Japan	26,955,418	2.02	94.3	23.6	99,858	9,282
Z2-1	*Eleusine coracana*	Japan	61,046,434	4.58	91.5	75.5	298,991	24,550
Br48	*Triticum aestivum*	Brazil	56,563,846	4.24	90.8	64.7	264,906	20,345
Br58	*Avena sativa*	Brazil	53,510,190	4.01	93.3	63.3	265,629	21,900
Dig41	*Digitaria sanguinalis*	Japan	60,124,854	4.51	29.6	6.81	488,052	13,392

The number of SNPs and indels between each of the tested isolates and 70-15 strain are shown.

Table S4. The threshold dN/dS for detecting outliers in each pairwise comparison between the representative isolates of *M. oryzae* when dN/dS = 99 was included.

		Subject				
		Ina168 (Oryza isolate)	GFSI1-7-2 (Setaria isolate)	Z2-1 (Eleusine isolate)	Br48 (Triticum isolate)	Br58 (Avena isolate)
Gene model	Ina168		99	0.116	1.697	1.800
	GFSI1-7-2	99		3.324	1.528	1.601
	Z2-1	2.256	1.838		1.469	1.505
	Br48	3.013	2.3	4.185		1.298
	Br58	2.848	2.107	4.098	1.218	
	70-15	99	99	1.325	1.664	1.594

The threshold values were calculated as the third quartile of dN/dS + 1.5 x interquartile ranges.

Table S5. The threshold dN/dS for detecting outliers in each pairwise comparison between the representative isolates of *M. oryzae* when dN/dS = 99 was excluded.

		Subject				
		Ina168 (Oryza isolate)	GFSI1-7-2 (Setaria isolate)	Z2-1 (Eleusine isolate)	Br48 (Triticum isolate)	Br58 (Avena isolate)
Gene model	Ina168		0.970	0	0.754	0.744
	GFSI1-7-2	0.994		1.219	0.729	0.746
	Z2-1	0.916	0.844		0.718	0.702
	Br48	0.919	0.881	1.231		0.705
	Br58	0.874	0.848	1.235	0.700	
	70-15	0.600	0.982	0.898	0.918	0.930

The threshold values were calculated as the third quartile of dN/dS + 1.5 x interquartile ranges. The threshold values of all the pairs were less than one except when Z2-1was used as a subject. dN/dS = 1.5 was used as the threshold value when dN/dS = 99 was excluded.

Table S6. Nucleotide sequences of transposable elements used in this study

Name	Accessions	Reference
Pot2	Z33638.1	[1]Kachroo et al. 1994[1]
Pot3	U60989.1	[2]Farman et al. 1996
MAGGY	L35053.1	[3]Farman et al. 1996
MGRL-3	AF314096	[4]Kang et al. 2001
MGL/MGR583	AF018033	[5]Hamer et al. 1989
Pyret	AB062507.1	[6]Nakayashiki et al. 1989
Inago 1	AB334124.1	[7]Sanchez et al. 2011
Inago 2	AB334125.1	Sanchez et al. 2011
SINE	U35313	[8]Kachroo et al. 1995
MINE-A	EF585235.1	[9]Gogvadze et al. 2007
MINE-B	EF585236.1	Gogvadze et al. 2007
MINE-C	EF585237.1	Gogvadze et al. 2007
MINE retrotransposon	AJ851229.1	[10]Fudal et al. 2005
Occan	AB074754.1	[11]Kito et al. 2003
Tf2	AY849688.1	-

[1]Kachroo,P., Leong,S.A. and Chattoo,B.B. Mol. Gen. Genet. 245 (3), 339-348 (1994)

[2]Farman,M.L., Taura,S. and Leong,S.A. Mol. Gen. Genet. 251 (6), 675-681 (1996)

[3]Farman,M.L., Tosa,Y., Nitta,N. and Leong,S.A. Mol. Gen. Genet. 251 (6), 665-674 (1996)

[4]Kang,S. Fungal Genet. Biol. 32 (1), 11-19 (2001)

[5]Hamer JE, Farrall L, Orbach MJ, Valent B, Chumley FG. Proc. Natl. Acad. Sci. U.S.A. 1989;86(24):9981-9985.

[6]Nakayashiki,H., Matsuo,H., Chuma,I., Ikeda,K., Betsuyaku,S., Kusaba,M., Tosa,Y. and Mayama,S. Nucleic Acids Res. 29 (20), 4106-4113 (2001)

[7]Sanchez,E., Asano,K. and Sone,T. J. Gen. Plant Pathol. 77, 239-242 (2011)

[8]Kachroo,P., Leong,S.A. and Chattoo,B.B. Proc. Natl. Acad. Sci. U.S.A. 92 (24), 11125-11129 (1995)

[9]Gogvadze,E., Barbisan,C., Lebrun,M.H. and Buzdin,A. BMC Genomics 8, 360 (2007)

[10]Fudal,I., Bohnert,H.U., Tharreau,D. and Lebrun,M.H. Fungal Genet. Biol. 42 (9), 761-772 (2005)

[11]Kito,H., Takahashi,Y., Sato,J., Fukiya,S., Sone,T. and Tomita,F. Curr. Genet. 42 (6), 322-331 (2003)
