# Supplementary material for: Host specialization of the blast fungus Magnaporthe oryzae is associated with dynamic gain and loss of genes linked to transposable elements
Source: BMC Genomics. 2016 May 18;17:370. doi: 10.1186/s12864-016-2690-6 (PMC4870811; doi:10.1186/s12864-016-2690-6)
Supplement: Additional file 3: — This file contains Supplemental Figures S1 to S8 and their legends. (PDF 1373 kb) [file 12864_2016_2690_MOESM3_ESM.pdf]

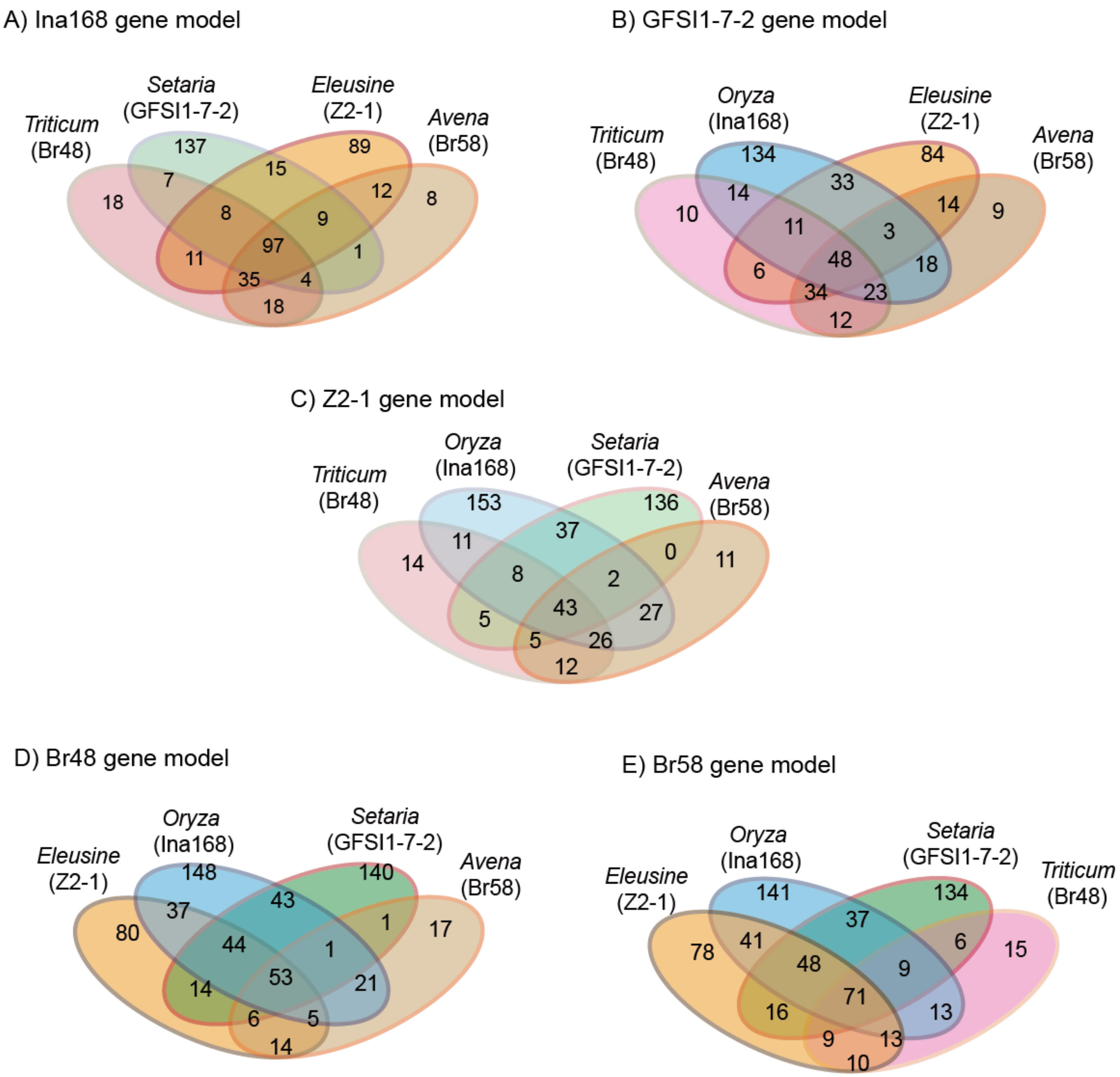

Figure S1. Venn diagrams of genes lost in the host-specific subgroups of *Magnaporthe oryzae*. The number of gene missing in each gene model was shown.

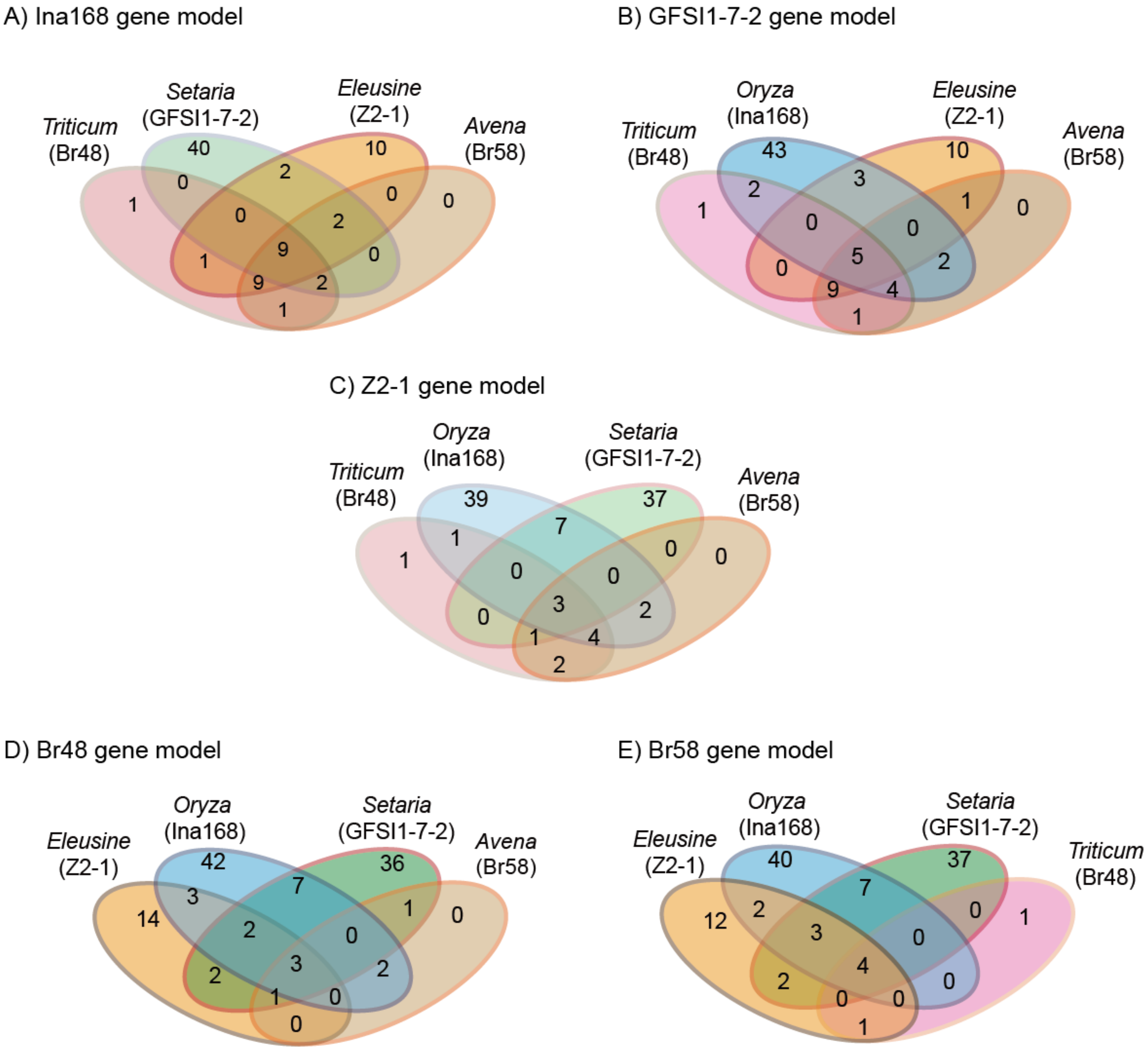

Figure S2. Venn diagrams of putative secreted protein genes lost in the host-specific subgroups of *Magnaporthe oryzae*. The number of gene missing in each gene model was shown

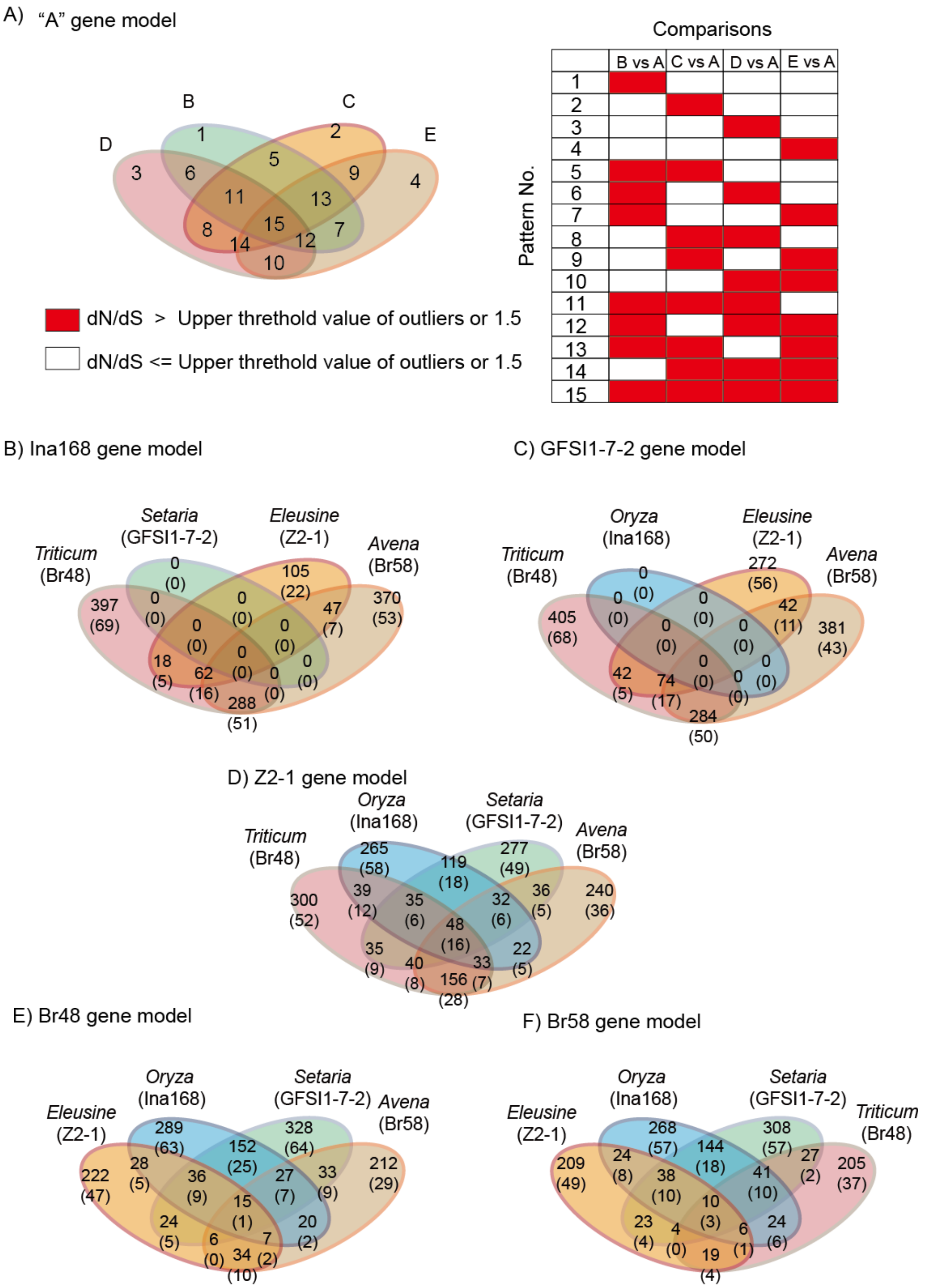

Figure S3. Venn diagrams of genes showing outliers of dN/dS including dN/dS = 99 between the reference and the other host-specific subgroups of *Magnaporthe oryzae*. (A) The relationship between the pattern of dN/dS outliers and the Venn diagrams is shown. According to the number of comparisons in which an outlier of dN/dS was detected, the pattern of dN/dS outliers was categorized. The number in the left Venn diagram are corresponding to the pattern number in the right table. (B)-(D) The Venn diagrams show the number of genes having outliers of dN/dS in each gene model. The number of secreted protein genes having outliers of dN/dS is shown in parenthesis.

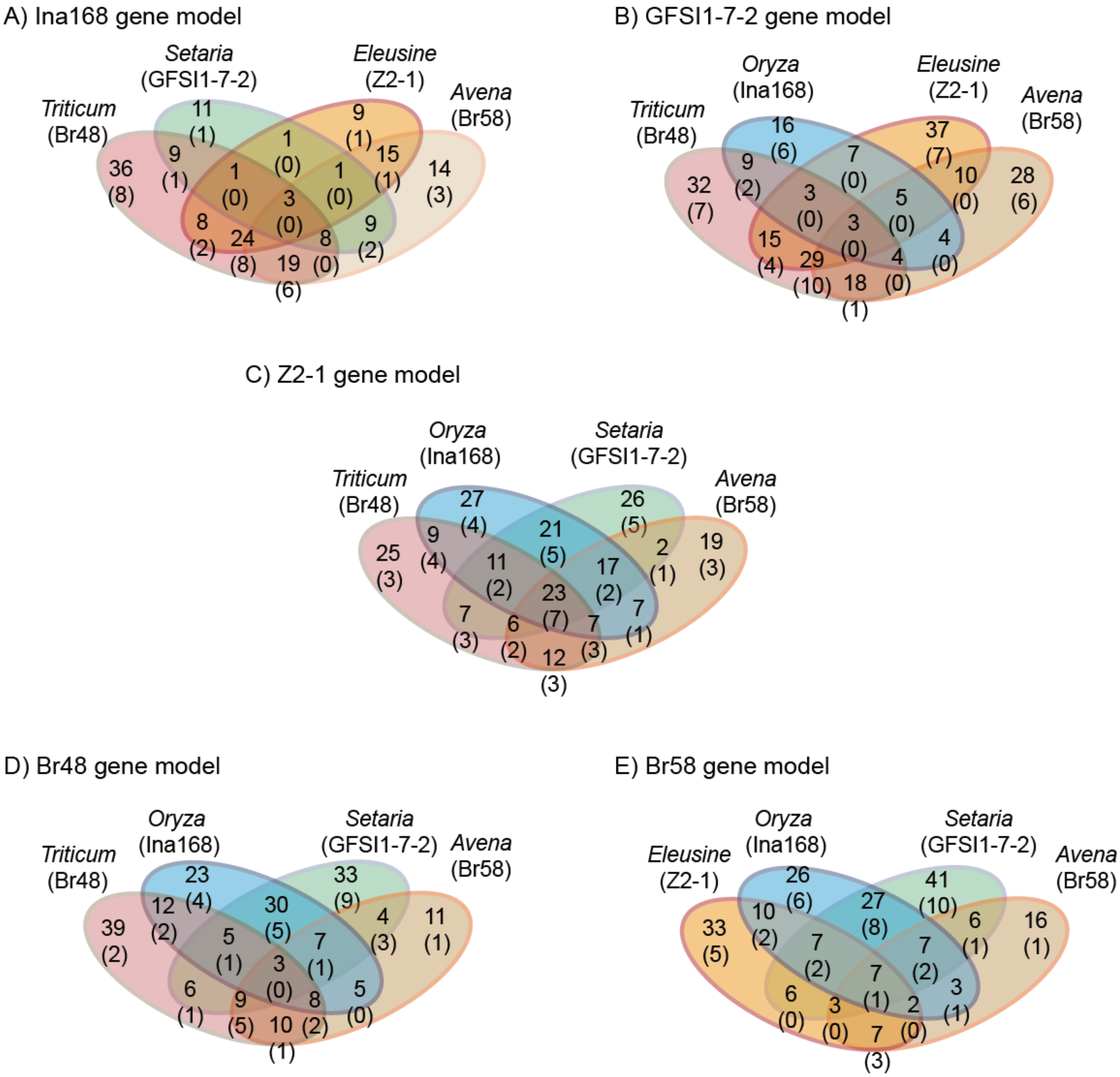

Figure S4. Venn diagrams of secreted protein genes showing outliers excluding dN/dS = 99 between the reference and the other host-specific subgroups of *Magnaporthe oryzae*. The threshold values for detecting outliers of dN/dS of all the pairwise comparisons are under 1.5. dN/dS = 1.5 was arbitrarily chosen as the threshold value. The Venn diagrams show the number of genes having dN/dS > 1.5 in each gene model. The number of secreted protein genes having dN/dS > 1.5 is shown in parenthesis.

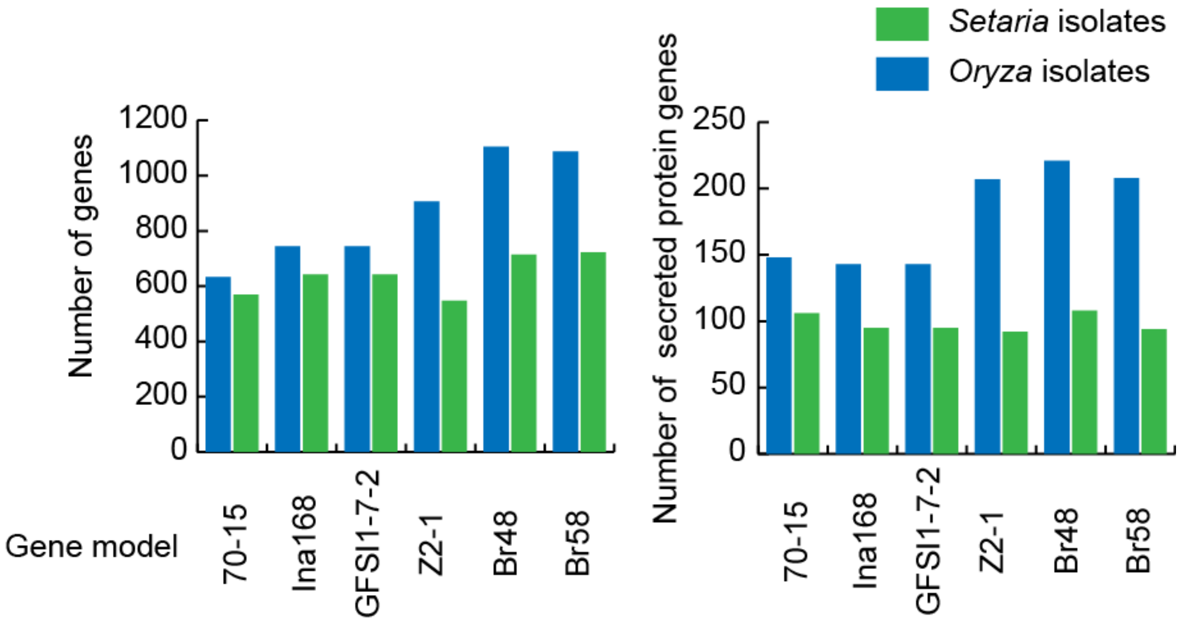

Figure S5. Presence/absence polymorphisms in four *Oryza* isolates and four *Setaria* isolates. The bar plot indicates the number of genes and secreted protein genes showing presence/absence polymorphisms. The short reads from four *Oryza* isolates and four *Setaria* isolates were aligned to the seven reference genome sequences. Presence/absence polymorphisms were estimated based on the breadth coverage over gene for each gene model of the reference genome. In any gene models, the number of genes showing presence/absence polymorphisms in *Oryza* isolates was larger than that in *Setaria* isolates.

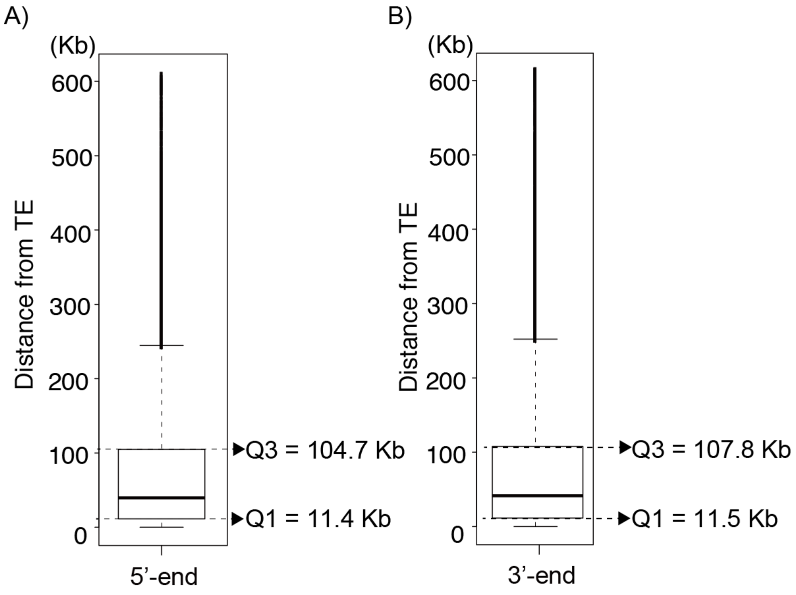

C)

	5'-end	3'-end
C1	< 11.4 Kb	< 11.5 Kb
C2	>= 11.4 Kb<br><= 104.7 Kb	>= 11.5 Kb<br><= 107.8 Kb
C3	> 104.7 Kb	> 107.8 Kb

Figure S6. Distribution of distance from 5-'end and 3'-end of genes to transposable elements (TEs). Box plots for the distance from 5'-end of genes to TEs (A) and the distance from 3'-end of genes to TEs (B) are shown. The median distance is 39.7 Kbp and 41.3 Kbp, respectively. Q1 and Q3 are 1st and 3rd quartiles of the distance, respectively. (C) The three categories of the distance from 5'-end and 3'-end of genes to TEs were shown. These categories were used for Figure 6C and Figure S8.

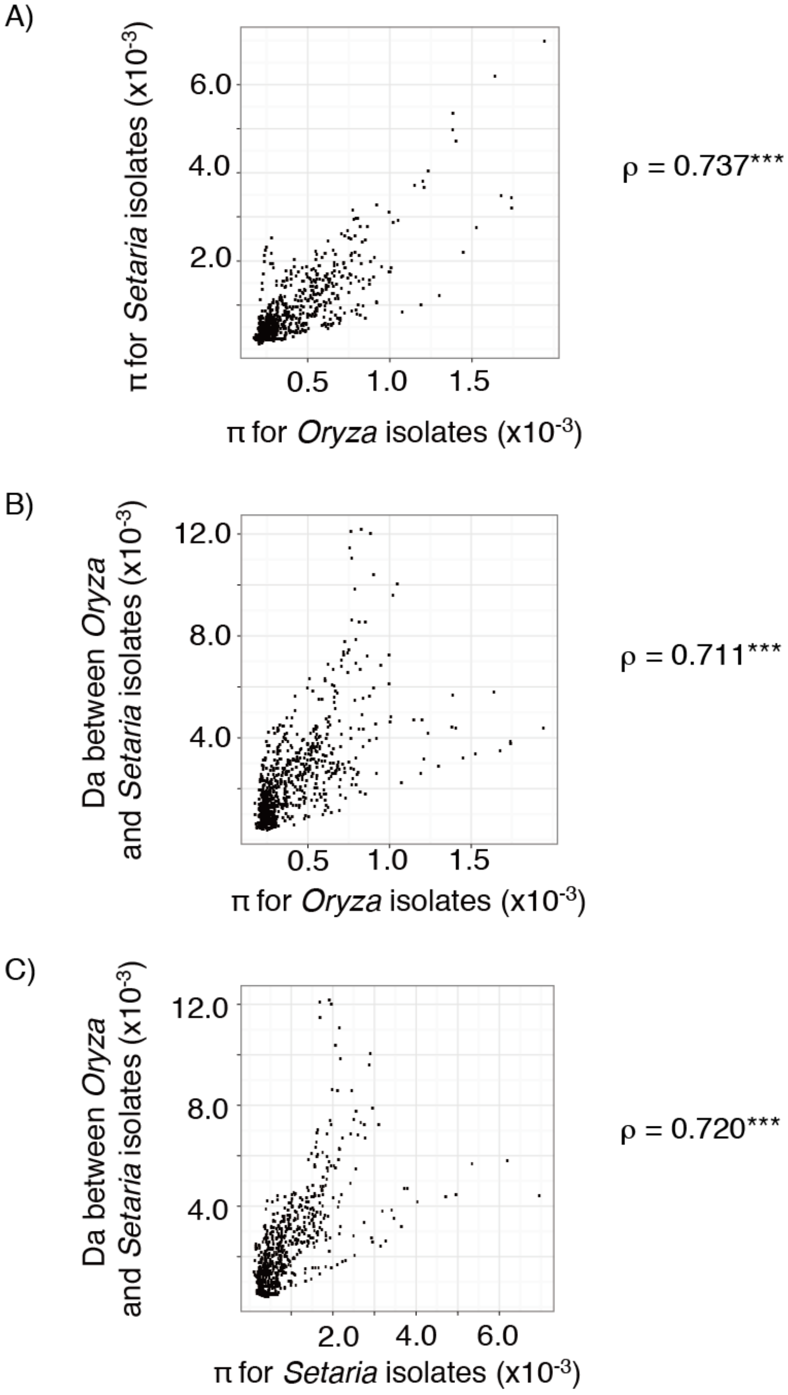

Figure S7. Correlation between *Oryza* π and *Setaria* π, between *Oryza* π and *Oryza-Setaria* divergence (*Da*), and between *Setaria* π and *Oryza-Setaria* divergence (*Da*).

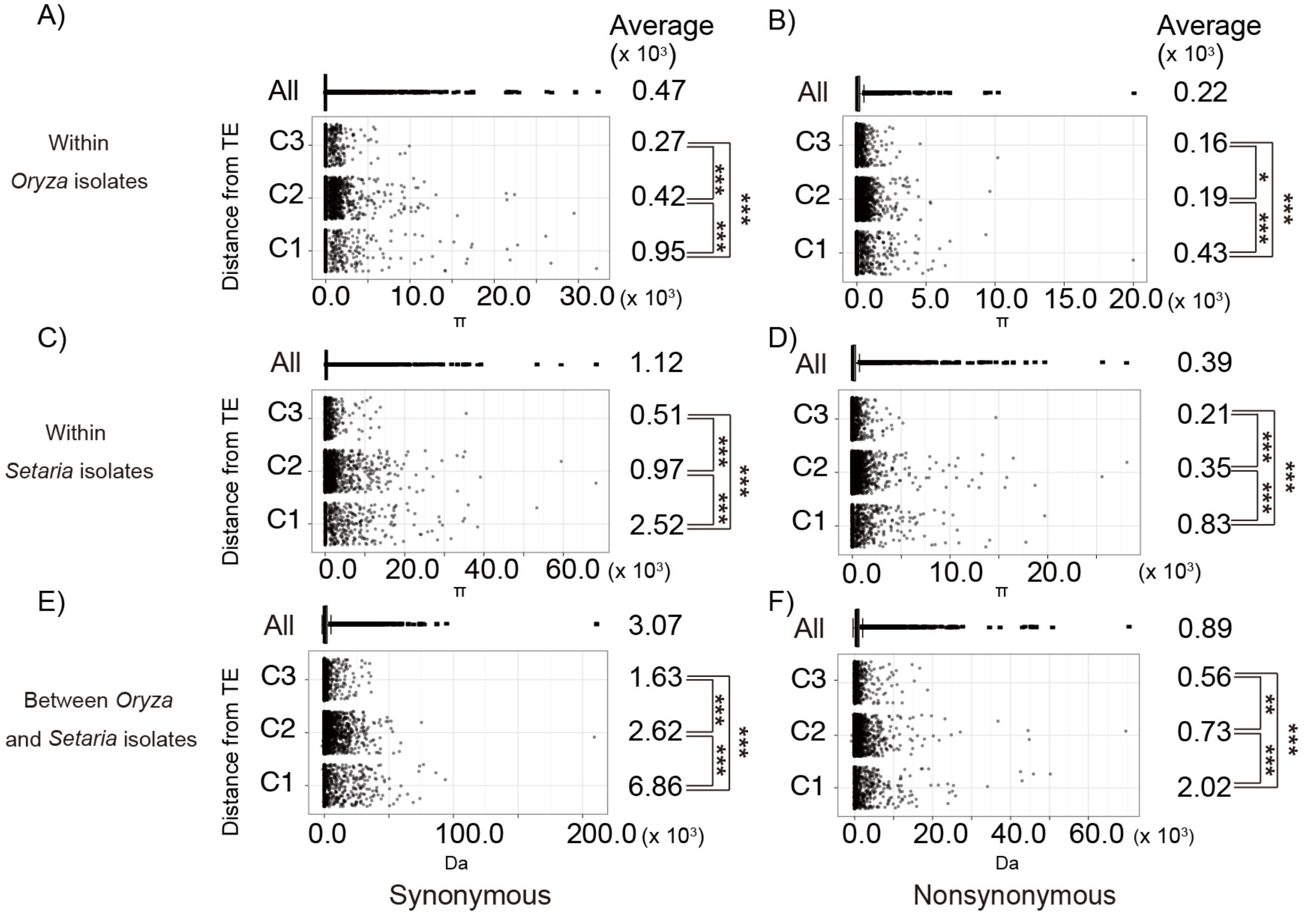

Figure S8. Relationship between transposable elements and levels of DNA polymorphisms of linked genomic regions.

Distribution of nucleotide variations according to distance from 5'-end and 3'-end of genes to TE. The panels from top to bottom are corresponding to synonymous π (π_{syn}) (A) and nonsynonymous π (π_{nonsyn}) (B) for *Oryza* isolates, π_{syn} (C) and π_{nonsyn} (D) for *Setaria* isolates, and synonymous Da (Da_{syn}) (E) and nonsynonymous Da (Da_{nonsyn}) (F) between *Oryza* and *Setaria* isolates. A box plot on the top of each panel indicates distribution of nucleotide variations for all of the categories C1 (The distance from 5'-end and 3'-end of genes to TE was less than 1st quartile), C3 (The distance was larger than 3rd quartile), and C2 (The distance ranged between C1 and C2). Outliers (> 1.5 x interquartile range + 3rd quartile) are shown as black circles. Jitter plots under the box plot of each panel indicate distribution of nucleotide variations for each of C1, C2, and C3. At the right side of each panel, average values of π/Da are shown. Statistical significance was evaluated by using Welch Two Sample t-test (*:P < 0.05, **:P < 0.01, and ***: P <0.001).
